# Supplementary material for: Donkey and Hybrid Anaesthetic Mortality in an Observational, Prospective, Multicentre Cohort Study
Source: Animals (Basel). 2025 Jun 25;15(13):1880. doi: 10.3390/ani15131880 (PMC12248588; doi:10.3390/ani15131880)
Supplement: Supplementary file 1 [file animals-15-01880-s001.zip › Supplementary Material S1 - instructions.pdf]

CEPEF4 v 4.1

INSTITUTION HOSPITAL ○ FIELD ○ DATE CASE

DIP. ☐ RES. ☐ PhD ☐ INT. ☐ VET ☐ TECH. ☐ Supervisor Supervised

SPECIES horse ○ pony ○ donkey ○ mule ○ SEX male ○ fem. ○ castr. ☐ preg. ☐

BREED AGE Y M D WEIGHT thin ○ normal ○ fat ○

ASA I ○ II ○ III ○ IV ○ V ○ E ☐ SCHEDULED scheduled ○ non sched. ○ urgent ○

REASON PROCED. diag ☐ colic ☐ abd. ☐ fract. ☐ ortho. ☐ urogen. ☐ ENT ☐ misc. ☐

TYPE TIVA ○ Inh. ○ PIVA ○ Standing sed. ○ TIMETABLE Normal ○ Out of hours ○

MONITORING ECG ☐ SpO_2 ☐ PAinv ☐ $EtCO_2$ ☐ FiO_2 ☐ Spirom. ☐ ABG ☐ Lact. ☐ Vol. Ag. ☐ T° ☐ NIBP ☐

Arterial line success ○ attempted ○ not attempted ○ Jugular vein catheter success ○ attempted ○ not attempted ○

	PREM	IND	MAIN	CRI	POST		PREM	IND	MAIN	CRI	POST
**ACP**	☐		☐	☐	☐	**KET**		☐	☐	☐	☐
**XYL**	☐		☐	☐	☐	**THIO**		☐	☐	☐	☐
**DET**	☐		☐	☐	☐	**PROP**		☐	☐	☐	☐
**ROM**	☐		☐	☐	☐	**AFX**		☐	☐	☐	☐
**MED**	☐		☐	☐	☐	**HAL**		☐	☐		
**DEX**	☐		☐	☐	☐	**ISO**		☐	☐		
**MOR**	☐		☐	☐	☐	**SEVO**		☐	☐		
**METH**	☐		☐	☐	☐	**DESF**		☐	☐		
**BUP**	☐		☐	☐	☐	**LIDO**	☐			☐	☐
**BUT**	☐		☐	☐	☐	**PBZ**	☐		☐		☐
**PET**	☐		☐		☐	**FLX**	☐		☐		☐
**FEN**	☐		☐	☐	☐	**MLX**	☐		☐		☐
**MDZ**	☐	☐	☐	☐	☐	**DOBU**				☐	☐
**DZP**	☐	☐	☐	☐	☐	**PHENYL**			☐	☐	☐
**GGE**		☐	☐	☐			☐	☐	☐	☐	☐

FASTED (h) food water HOSP. before ☐ after ☐ INDUCTION free ○ assist. ○ gate ○ sling ○ table ○

LOCOREGIONAL No ○ Yes ○ Epidural ☐ Spinal ☐ Block ☐ Lido. ☐ Bupi ☐ Ropi. ☐ Morp. ☐ other ☐

FLUIDS No ○ Yes ○ Crystall. ☐ Colloids ☐ Hyper. ☐ Blood ☐ other ☐ ATB

O_2 ☐ AIR ☐ N_2O ☐ INTUBATION Yes ○ No ○ Diff. ☐ CIRCUIT circle ○ none ○ other

MECHANICAL VENTILATION No ○ Yes ○ VCV ☐ PCV ☐ PEEP ☐ CPAP ☐ NMBA ☐

COMPLICATIONS Hypotension ☐ Hypox. ☐ Hypotherm. ☐ Hypercap. ☐ Arrhythm. ☐ Bleeding ☐ Excitation ☐ Intra. awake ☐

EMERG DRUGS Atrop. ☐ Adren. ☐ Ephed. ☐ Nora. ☐ Neostig. ☐ Phenyl. ☐ other ☐

RECUMB. TABLE LL ☐ RL ☐ D ☐ DURATION ANAE (min) SURG (min) REC. free ○ ropes ○ manual ○

best → worst

REC. SCORE 1 ○ 2 ○ 3 ○ 4 ○ 5 ○ REC. TIME (min) TUBE REC NO ○ orotrach. ○ nasal ○ O_2 ☐

DEATH NO ○ YES ○ EUT. ○ No ○ Prem. ○ Ind. ○ Main. ○ Rec. ○ <12 h ○ >12 h ○ when (days)

PRE-EXISTING CONDITIONS

POSTOPERATIVE CARE

COMMENTS (reason of death, medical treatments, emergencies, other complications, etc.).

RESET SEND

The Confidential Enquiry into Perioperative Equine Fatalities

INSTRUCTIONS FOR FILLING THE CEPEF4 QUESTIONNAIRE

Filling this form is very easy. We are asking you to fill in a form for EVERY patient (horse, pony, donkey or mule). All cases must be included or the data will be biased and will give false information about the equine anaesthetic death and complication rate. This means that the form is filled in for every case undergoing ***general anaesthesia*** (all short procedures in which general anaesthesia is induced should be included). ***Also, any standing sedations for surgery, magnetic resonance imaging (MRI), computer tomography (CT) and scintigraphy and requiring a constant rate infusion (CRI) or at least one top-up for maintenance*** (please, do not include sedations in which only one injection was required or for any other procedures, such as radiography, sedation for intravenous fluid or placing a nasogastric tube, etc.).

The forms are in pdf format. You have to use Adobe Acrobat to fill in them, a free app that you can download onto your computer or smartphone/tablet by clicking on these links for iPhone or Android. When the case is finished, the form will automatically be sent by email by pressing the button "SEND". Then, your mail app will open, and the form will appear as an attachment. Then, you send the e-mail to the e-mail address previously linked cepef4@gmail.com. You can see some self-explanatory videos at https://cepef4.wordpress.com/videos.

Please, find below some comments/explanations in order to standardise data collection.

DESCRIPTION OF THE SECTIONS (from top to bottom)

INSTITUTION: fill in the name of the veterinary clinic or hospital in which the anaesthesia / standing sedation was performed. Alternatively, the name of the person who carried out the anaesthesia / standing sedation can be used (e.g. private practitioner).

FACILITIES: this section gives you the opportunity to indicate whether the procedure was performed in the "HOSPITAL" facilities or under "FIELD" conditions.

DATE: fill in the date when the procedure took place.

CASE: case identification number. If you wish, you can enter your cases numbered sequentially in order to preserve privacy and anonymity.

WHO DID THE ANAESTHESIA / STANDING SEDATION: tick the qualification of the people who performed the anaesthesia. Abbreviations are: "DIP.", for Diplomate certified by the ECVAA or ACVAA; "RES.", for ECVAA or ACVAA resident or other recognized anaesthesia specialist training programme; "INT.", for postgraduate veterinarians doing an internship regardless of the speciality or a residency other than Veterinary Anaesthesia (e.g. residency in Equine Surgery); "VET.", for veterinary surgeon without specialization; "TECH.", for veterinary technicians or nurses.

For the "Supervisor" and "Supervised" boxes use the initials of the people involved in a consistent way [e.g. an ECVAA Diplomate (LS) supervising a resident (HM)]. If just one person, fill in the initials in the box "Supervisor" only.

SPECIES: tick horse, pony, donkey or mule. Pony's height limits (e.g. 148 cms) will be considered according to each country's breed societies.

If a patient dies, please contact cepef4@gmail.com. This information will be treated as strictly confidential.

SEX: tick “male” for male or “fem.” for female. If the male is castrated tick “castr.”. If the mare is pregnant, please tick “preg.”. If pregnant, include in the section “Comments” at the bottom of the questionnaire whether in the initial, middle or final third of gestation.

BREED: fill in the breed of the horse/pony/donkey/mule.

AGE: with years, months and/or days.

WEIGHT: fill in weight in kg with no decimal places.

BCS (body condition score): tick the most appropriate for the patient.

ASA: based on the classification for humans of the American Society of Anesthesiologists.

- ASA I: normal healthy animal, no underlying disease (e.g. healthy horse undergoing castration).
- ASA II: minor disease present. Animal with slight to mild systemic disturbance, animal able to compensate (e.g. healthy horse undergoing bilateral stifle arthroscopy).
- ASA III: obvious disease present. Animal with moderate systemic disease or disturbances, mild clinical signs. (e.g. horse with compensated atrial fibrillation, anemic patient which does not need transfusion).
- ASA IV: significantly compromised by disease. Animals with preexisting systemic disease or disturbances or a severe nature (e.g. colic horse with moderate pain due to colon impaction presented with dehydration, mild signs of hypotension, etc.).
- ASA V: Moribund. Surgery often performed in desperation on animal with life threatening systemic disease (e.g. colic horse with severe pain due to colic torsion, presented with severe dehydration and hypotension, signs of shock, etc.).

If the procedure is an emergency, please, tick the box “E”.

SCHEDULED: tick one of the three options for the procedure: “scheduled” for elective surgeries, “no sched.” for elective surgeries not scheduled for the day and not urgent (e.g. horses arriving in the morning for a lameness and then suddenly the surgeon comes and asks: *“could you quickly do an anaesthetic?”.* We believe that this is not ideal but is a real clinical scenario). Finally, “urgent” when the procedure needs to be performed without being scheduled, as it is considered an emergency.

REASON: describe briefly the reason for anaesthesia/standing sedation (e.g. “bilateral stifle arthroscopy”, “colic/exploratory laparotomy”, “castration”, “eye enucleation”, “tie-back”, “dental extractions”, etc.).

PROCED.: classification of the procedure.

- “diag” for diagnostic purposes (e.g. CT, MRI, scintigraphy, etc.).
- “colic” for colic surgery (e.g. colic undergoing exploratory laparotomy, enterectomy, colon displacement, colon torsion, etc.)
- “abd.” for other abdominal surgeries. Procedures requiring a laparotomy (e.g. ovariectomy under standing sedation, umbilical hernia under general anaesthesia, etc.).
- “fract.” for limb fractures (e.g. condylar fracture, P1 fracture, ostheosynthesis for fractures, etc.).
- “ortho.” for other general orthopaedic procedures (e.g. arthroscopy, periosteal stripping in a foal, desmotomies, inter-spinous ligament desmotomy, etc.)
- “urogen.” for procedures that involve the urinary tract and genitalia (e.g. castration, rectovaginal fistula, etc.).
- “ENT” for ear-nose-throat procedures (e.g. tie-back, ethmoid hematoma, etc.).
- “misc.” for miscellaneous, other procedures (e.g. dental procedures, wound debridement/treatment, eye enucleation, skin surgery, cast change, sarcoid removal, mandibular fracture, etc.).

If a patient dies, please contact cepef4@gmail.com. This information will be treated as strictly confidential.

TYPE (for protocol type):

- "TIVA" for total intravenous anaesthesia: if anaesthesia was induced and maintained using ***ONLY*** intravenous drugs.
- "Inh." for inhalational anaesthesia: if maintenance was performed ***ONLY*** using volatile agents. Anaesthesia could be induced with either intravenous drugs or volatile agents.
- "PIVA" for partial intravenous anaesthesia: if anaesthesia was maintained using volatile agents together with constant rate infusions (CRIs) (e.g. romifidine, lidocaine, ketamine, etc…). Anaesthesia could be induced with either intravenous drugs or volatile agents.
- "Standing sed." for surgical or diagnostic procedures under standing sedation lasting longer than 30 minutes requiring at least one top-up or infusion to prolong sedation: for procedures performed with the patient sedated, with no induction of general anaesthesia.

TIMETABLE: "Normal" if the procedure ***started within the normal working hours*** of the institution/hospital, or "out of hours", if the procedure ***started after the normal working hours***.

MONITORING: tick if any of the following were used: tick all that apply

- "ECG": for electrocardiogram.
- "SpO_2": for pulse oximetry.
- "PAinv": for invasive arterial pressure.
- "$EtCO_2$": for monitoring carbon dioxide with capnography.
- "FiO_2": for monitoring the inspiratory fraction of oxygen.
- "Spirom.": for spirometry.
- "ABG": for arterial blood gas samples taken for analysis.
- "Lact.": for measurement of blood lactate.
- "Vol. Ag.": for volatile agents measured for inspired fraction and end-tidal concentration.
- "T°": for measurement of peripheral body temperature (e.g. rectal, oseophageal).
- "NIBP": for non-invasive blood pressure.

Arterial line and jugular catheter: if the placement of a catheter was successful, therefore "success", "attempted" or "not attempted" (e.g. facial artery for arterial line and jugular vein for central line).

ANAESTHETIC PROTOCOL: tick the drugs used and in which phase: premedication "PREM", induction "IND", maintenance "MAIN", constant rate infusions "CRI" during maintenance and/or the immediate postoperative period "POST". When a horse receives a CRI during maintenance, only the drug under the column "CRI" has to be ticked. When top-ups are administered (e.g. ketamine, morphine, lidocaine, phenylephrine), the drug must be ticked under the column "MAIN". If a drug was used in several phases, please, tick all the phases in which the drug was used.

Abbreviations used in this section are: "ACP", acepromazine; "XYL", xylazine; DET, detomidine; "ROM", romifidine; "MED", medetomidine; "DEX", dexmedetomidine; "MOR", morphine; "METH", methadone; "BUP", buprenorphine; "BUT", butorphanol; "PET", pethidine; "FEN", fentanyl; "MDZ", midazolam; "DZP", diazepam; "GGE", glyceryl guaiacole ether; "KET", ketamine; "THIO", thiopental; "PROP", propofol; "AFX", alfaxalone; "HAL", halothane; "ISO", isoflurane; "SEVO", sevoflurane; "DESF", desflurane; "LIDO", lidocaine; "PBZ", phenylbutazone; "FLX", flunixin; "MLX", meloxicam; "DOBU", dobutamine; "PHENYL", phenylephrine (PHENYL when given IV as a bolus is to be included as "MAIN", when as an infusion as "CRI" and "POST" intranasally for the recovery). Please note than the last row in the second column of drugs is free for inclusion of any other drug used in the anaesthetic protocol in any of the different phases

If a patient dies, please contact cepef4@gmail.com. This information will be treated as strictly confidential.

(tick the corresponding box to choose the correct phase). Emergency drugs should be included in the specific section below.

FASTED: fill in the number of hours the animal was deprived of food and water. Write "0" if no deprivation occurred. If impossible to estimate, leave this blank.

HOSP.: tick if the horse stayed at the hospital "before" and "after" the surgery. Do not tick "before" if the horse came to the institution and immediately went for surgery, and do not tick "after" if the horse was immediately discharged.

INDUCTION: tick "free" if the induction was without assistance or with head restraint only, "assist." if supported against the wall by two or more people, using a swing "gate", a "sling" or using a tilted "table".

LOCO-REGIONAL: For use of any loco-regional technique, tick "No" or "Yes". If "Yes", tick "epidural", "spinal" or any other type of "block". Fill in the space describing the technique (e.g. peribulbar block, mandibular block, for epidural "Co1-Co2", etc…). Also, the name of the drug (e.g. "Lido." for lidocaine, "Bupi" for bupivacaine, "Ropi." for ropivacaine, "Morp." for morphine or "other". If "other", include this in the section "COMMENTS" at the bottom of the questionnaire).

FLUIDS: For fluid administration tick "No" or "Yes". If "Yes", tick all that apply of "Crystall." for isotonic crystalloids, "Colloids", "Hyper." for hypertonic saline (e.g. 7.2%), "Blood" or "other". Name "other" in the space if you use any other type of fluid.

ATB: fill in the antibiotics administered as premedication and/or during the procedure, and the route of administration.

O_2: tick if oxygen was administered.

AIR: tick if air was administered. Tick "O_2" and "air" if both were used.

N_2O: tick if nitrous oxide was administered.

INTUBATION: tick if the trachea was intubated "Yes" or not "No". If intubation was difficult, then tick "Diff.".

CIRCUIT: tick "circle" if a circle circuit was employed, "none" (e.g. TIVA under field conditions) or "other" (e.g. To and Fro).

MECHANICAL VENTILATION: tick "No" if the horse breathed spontaneously throughout. Tick "yes" for other modes of ventilation: "VCV" (Volume Controlled Ventilation), "PCV" (Pressure Controlled Ventilation), PEEP" (Positive End Expiratory Pressure) or "CPAP" (Continuous Positive Airway Pressure).

NMBA: tick if neuromuscular blocking agents were employed and, if ticked, write the name in the space.

COMPLICATIONS:

- "Hypotension" if mean arterial pressure was lower than 70 mmHg for more than 15 consecutive minutes, or that required treatment.
- "Hypox." for hypoxaemia. If partial pressure of oxygen (PaO_2) was lower than 60 mmHg or when "desaturation" occurred (SpO_2 lower than 90%) for more than 15 minutes, or that required treatment.
- "Hypotherm." for hypothermia. If peripheral body temperature (e.g. rectal, oseophageal) was lower than 35°C for more than 15 minutes, or that required treatment.
- "Hypercap." for hypercapnia. If partial pressure of carbon dioxide or $EtCO_2$ was higher than 65 mmHg for more than 15 consecutive minutes, or that required treatment.
- "Arrhythm." for arrhythmias. If any relevant arrhythmia was observed. For example, ventricular tachycardia requiring lidocaine for treatment. Second degree atrioventricular blocks after administration of an alpha-2 agonist without haemodynamic effects should not be included.

If a patient dies, please contact cepef4@gmail.com. This information will be treated as strictly confidential.

- “Bleeding” if any bleeding occurred, requiring treatment such as fluid infusion (e.g. colloids, blood transfusion) or surgical ligature.
- “Excitation” while premedicated before any procedure, during standing sedation or during the induction/recovery phases of general anaesthesia leading to risk of injury to personnel, the patient itself or the equipment.
- “Intra. awake” for the plane of anaesthesia becoming too light, with nystagmus or movement requiring additional treatment (e.g. extra doses of ketamine or thiopental).

EMERG DRUGS: tick if any intravenous emergency drugs were employed: “Atrop.” for atropine, “Adren.” for adrenaline/epinephrine, “Ephed.” for ephedrine, “Nora.” for noradrenaline/norepinephrine, “Neostig.” for neostigmine, “Phenyl.” for phenylephrine, and “other”. If “other”, name the drug in the space (e.g. atipamezole).

REC: for recumbency of the animal on the table. Tick “LL” for left lateral, “RL” for right lateral, or “D” for dorsal recumbency. Please note that the boxes are not exclusive (e.g. you can tick two boxes for a horse that was moved from RL to dorsal).

DURATION: in minutes (min) of anaesthesia (ANAE), from induction of general anaesthesia (or initial bolus for standing sedation) until disconnection from the anaesthetic machine (or the end of infusion or top-ups for standing sedation). Surgery (SURG) time in minutes (min) from first incision until the last stitch.

REC.: for the type of recovery used. Tick “free” if the recovery was without assistance, “ropes” if assisted with ropes or “manual” as for instance in foals or donkeys. If any other methods such as a pool recovery, slings or helicopter net were used, include them in the “COMMENTS” section.

REC. SCORE: to score recovery qualities. Please use the following Simple Descriptive Scale:

1) One attempt to stand, no ataxia
2) One to two attempts to stand, some ataxia
3) >2 attempts to stand but quiet recovery
4) >2 attempts to stand, excitation
5) Severe excitation. Patient injured

REC. TIME: (from general anaesthesia) in minutes**.** The duration of recovery from the end of general anaesthesia until the patient stands up.

TUBE REC.: if a tube was used or not in during recovery. Tick “no” if no tube was in place throughout recovery. If an orotracheal tube was left in place until the patient was standing choose “orotrach.”. Choose “nasal” if a nasotracheal/nasopharyngeal tube was placed after orotracheal extubation.

O_2: tick if oxygen was supplied during the recovery phase.

DEATH: this section implies ***two different questions***. ***First*** tick “NO”, if the patient was alive at the time of recovery, “YES” if dead and “EUT” if the animal had to be euthanised.

The ***second*** question asks when the patient died or when had to be euthanised. Tick “No” if the patient was alive at 7 days or has been discharged, “Prem.” if died during premedication, “Ind.” if died during induction of general anaesthesia, “Main.” if died during maintenance of general anaesthesia or standing sedation, or “Rec.” if died during recovery after general anaesthesia. Also, if death occurred during the postoperative period within 12 hours after induction of general anaesthesia (or initial bolus for standing sedation) “< 12”. If > 12 hours tick “> 12” and fill in the box the day that the patient died. If the horse/pony/donkey/mule was euthanized for medical or surgical reasons, please tick “EUT” and state the time of euthanasia.

If the patient dies, please contact cepef4@gmail.com. This will be strictly confidential.

If a patient dies, please contact cepef4@gmail.com. This information will be treated as strictly confidential.

PRE-EXISTING CONDITIONS: if any. Describe briefly.

POSTOPERATIVE CARE: brief description.

COMMENTS: please use this section as an opportunity to include any information you could not write in the questionnaire itself (e.g. suspected cause of death, preexisting diseases, other complications, previous medical treatments, emergency treatment, type of arrhythmia, nasotracheal intubation, postoperative pyrexia, etc.).

SEND: Press this button to send the form.

RESET: Press the button to erase the form and start a new case.
