# Supplementary material for: Donkey and Hybrid Anaesthetic Mortality in an Observational, Prospective, Multicentre Cohort Study
Source: Animals (Basel). 2025 Jun 25;15(13):1880. doi: 10.3390/ani15131880 (PMC12248588; doi:10.3390/ani15131880)
Supplement: Supplementary file 1 [file animals-15-01880-s001.zip › Supplementary Material S2 - 12-question checklist.pdf]

The 12 questions of the final checklist completed by MGM and the ambassador of each centre during the last round of online meetings to validate the data of each centre.

Q1 Did you complete a form for each general anaesthesia performed in your centre within a period of time? (*This allowed for holidays and staff absences; all cases within a specified period could be omitted).

Q2 Did you complete a form for each standing sedation performed in your centre within a period of time? (*Not all the centres sending information of cases of general anaesthesias were sending cases for standing sedations. The information on standing sedations will be reported in a separate publication).

Q3 Are there any errors remaining in the data of your centre?

Q4 Are there any duplicates remaining in the data of your centre?

Q5 Is there any "wrong" data in the data of your centre?

Q6 Does the number of cases of the report we sent you (Domenech et al. 2024) based on the data you sent us match with the control or logbook of your centre?

Q7 Are there any missing cases?

Q8 Does the number of fatalities of the report we sent you match with the ones of the logbook of your centre?

Q9 Have all the specific online surveys been completed for each NON-COLIC DEAD? (*The information on NON-COLIC DEADs will be reported in a separate publication).

Q10 Is the report we sent you consistent with the practice/protocols of your centre?

Q11 Did you sign both agreement forms? (*Initial agreement form – supplementary material S2A + final Data User Agreement form – supplementary material S2B).

Q12 Did you complete the online survey for the IT paper (Domenech et al. 2024)?
